# Organoids research progress in gynecological cancers: a bibliometric analysis

**DOI:** 10.3389/fonc.2024.1484074

**Published:** 2024-10-28

**Authors:** Baiyun He, Huihao Ma, Hongbo Yu, Dongmei Li, Li Zhang, Junjie Wang

**Affiliations:** ^1^ Department of Gynecology, Affiliated Renhe Hospital of China Three Gorges University, Yichang, China; ^2^ Department of Neurosurgery, The First Affiliated Hospital of Dalian Medical University, Dalian, Liaoning, China

**Keywords:** gynecological cancers (GC), organoids, bibliometric analysis, ovarian cancer (OC), endometrial cancer (EC), cervical cancer (CC)

## Abstract

**Background:**

Gynecological cancers (GC) pose a severe threat to the health and safety of women’s lives, and organoids, as *in-vitro* research models, have demonstrated significant advantages in simulating tissue characteristics and drug screening. In recent years, there has been a rapid increase in research outcomes related to organoids in GC. However, there has been no bibliometric study concerning.

**Methods:**

Publications related to GC and organoids from 2010-2023 were retrieved from the Web of Science Core Collection (WoSCC). We conducted a bibliometric analysis and visualization using CiteSpace, VOSviewer, and the Bibliometrix R Package. This analysis included the spatiotemporal distribution, author, sources, references, and keywords.

**Results:**

A total of 333 publications were included. The number of annual publications indicated an explosive phase of development since 2019. The USA was the most important country in terms of cooperation, publication output, citation and centrality. University of California system ranked first in productivity among institutions, and HIPPO Y is the most relevant author in the research field. *CANCERS* published the most documents, and *NATURE* is the most cited sources. Analysis of Keywords and References, it is possible to establish the trend, and find the hotspots in the research field.

**Conclusion:**

This bibliometric analysis delineated global landscapes and progress trends in GC organoids research. This study emphasized that organoids can effectively replicate the original tissue or tumors, providing a good *in-vitro* model for research on tumor-related mechanisms and showing significant advantages in drug screening and efficacy clinical prediction. Additionally, as preclinical models, they provide compelling evidence for personalized therapy and prediction of patient drug responses.

## 1 Introduction

Gynecological cancers(GC) represent a significant global health challenge due to their high prevalence and mortality rates (1, 2). The development of novel therapeutic strategies and early diagnostic tools is imperative to improve patient outcomes. In recent years, the advent of organoid technology has provided an ideal model *in-vitro* for cancer research (3). Organoids, as three-dimensional (3D) cell cultures that simulate the body’s tissues and organs *in vitro (* 4), include multiple cell types (5) and can faithfully reproduce the histology, genetics, and mutational profiles of the source tissue (4, 6). They offer a versatile tool for diagnostics, disease modeling, drug discovery, and personalized medicine (6). In addition, organoids serve as clinical models for tumors, offering an ideal model for foundational and clinical studies and bridging the gap between *in-vitro* studies, animal models, and clinical treatment responses in patients (7, 8).

Despite the rapid growth of organoids research in GC, particularly in ovarian cancer (OC), endometrial cancer(EC) and cervical cancer(CC), a comprehensive assessment of the scientific output, trends, and current hotspots in this area remains largely unexplored. Bibliometric analysis, a quantitative method for mapping and evaluating scientific literature, provides a systematic approach to understanding the structure, development, and impact of research fields over time (9, 10).

The objective of this study is to conduct a bibliometric analysis of the scholarly literature pertaining to the utilization of organoids in GC research. By examining the publication trajectories, authorship dynamics, institutional collaborations, and thematic shifts, the research endeavors to map the landscape of organoid research in this field. Furthermore, the study seeks to identify the most influential studies, prominent researchers, and key institutions driving the advancement of organoid technology in GC. We will also analyze the thematic shifts and find the hotspots by examining the keywords and references associated with organoid studies in GC. This analysis will not only provide insights into the current state of research but also highlight potential areas for future investigation.

## 2 Methods

### 2.1 Data collection

The Web of Science (WoS) is globally acknowledged as a highly credible citation database, as affirmed by a distinguished publisher (11). To enhance the representative and accessible data, our search encompassed both the Web of Science Core Collection (WoSCC) database and the Science Citation Index Expanded (SCI-EXPANDED). Our search query integrated Medical Subject Headings (MeSH) terms and keywords of “Organoids” with “ Endometrial cancers” OR “Ovarian cancer” OR “Cervical Cancers”. We covered a period from January 1, 2010, to April 30, 2024. The data retrieved were collected on May 12, 2024, to mitigate any potential biases due to daily updates. A total of 391 records were initially identified, which were then narrowed down to articles (n=253) and reviews (n=80), all of which were in English. These records were exported in full-text format, inclusive of cited references. Two authors independently accomplished all literature searches, and the outcomes were compared. Any differences were discussed with a third author to establish which articles would be incorporated in the ultimate analysis.

### 2.2 Data analysis

VOSviewer (v.1.6.20), CiteSpace (v.6.3.R1 Basic), and the Bibliometrix R Package were utilized to analyze and visualize the entire collection of 333 documents. The emergence and development of these bibliometric analysis software significantly propelled research and expanded applications in the field of information visualization (9).

VOSviewer, the Visualization of Similarities viewer, is a Java-based free software developed by Van Eck and Waltman at the Centre for Science and Technology Studies (CWTS), Leiden University, in 2009, designed for constructing and visualizing bibliometric networks (12). We used VOSviewer software to analyze and visualize the citation sources relationships, as well as the co-occurrence network of keywords and references.

CiteSpace, developed by Professor Chaomei Chen at Drexel University, is a multifaceted, time-sensitive, and dynamic citation visualization analysis software that focuses on uncovering latent knowledge within scientific literature, evolving within the context of scientometrics and information visualization (11, 13, 14). By employing CiteSpace software, we analyzed and visualized the network of countries/regions and institutions, the institutions and references with the strongest citation bursts, and the dual-map overly of journals. It extracted the publication counts of the top 10 institutions and calculate the centrality values.

**Figure 1:**
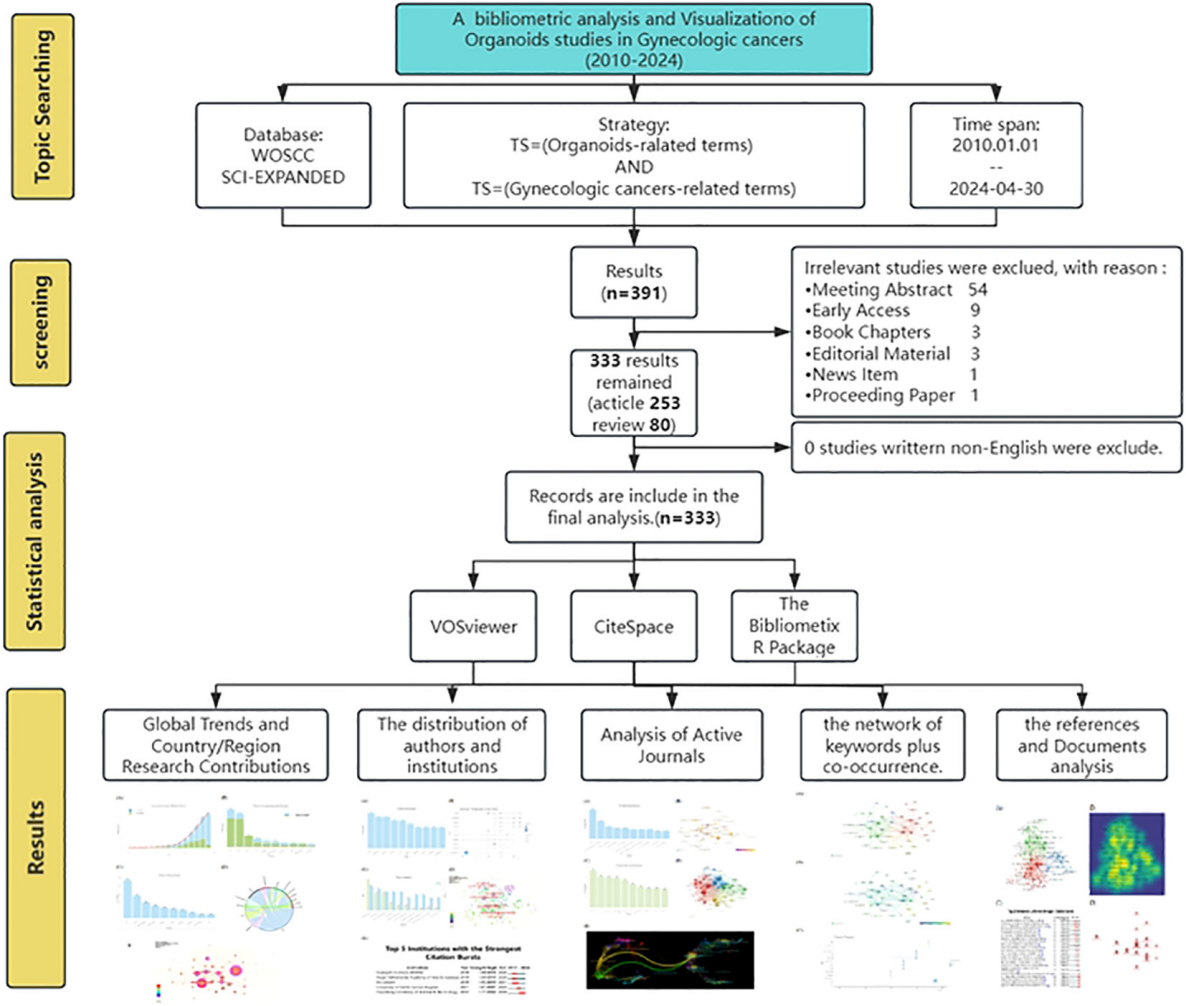
Flow diagram of the study identification and inclusion process.

The Bibliometrix R Package, an open-source and free toolkit for scientometric analysis and visualization, was co-developed by Italian statisticians Massimo Aria and Corrado Cuccurullo (9, 15). Using the Bibliometrix R package to extract annual and cumulative publication counts, as well as publication volumes by country/region, author, and journal, and then displaying these data with bar charts using the R language. The tool displayed the cooperation between countries/regions with a chord diagram, and shown the top 10 authors’ publications and the trend topics of keywords over time with a line char. **Figure 1** illustrates the flowchart of the search strategy and analysis process in this study.

## 3 Results

### 3.1 Global contribution to the field

**Figure 2:**
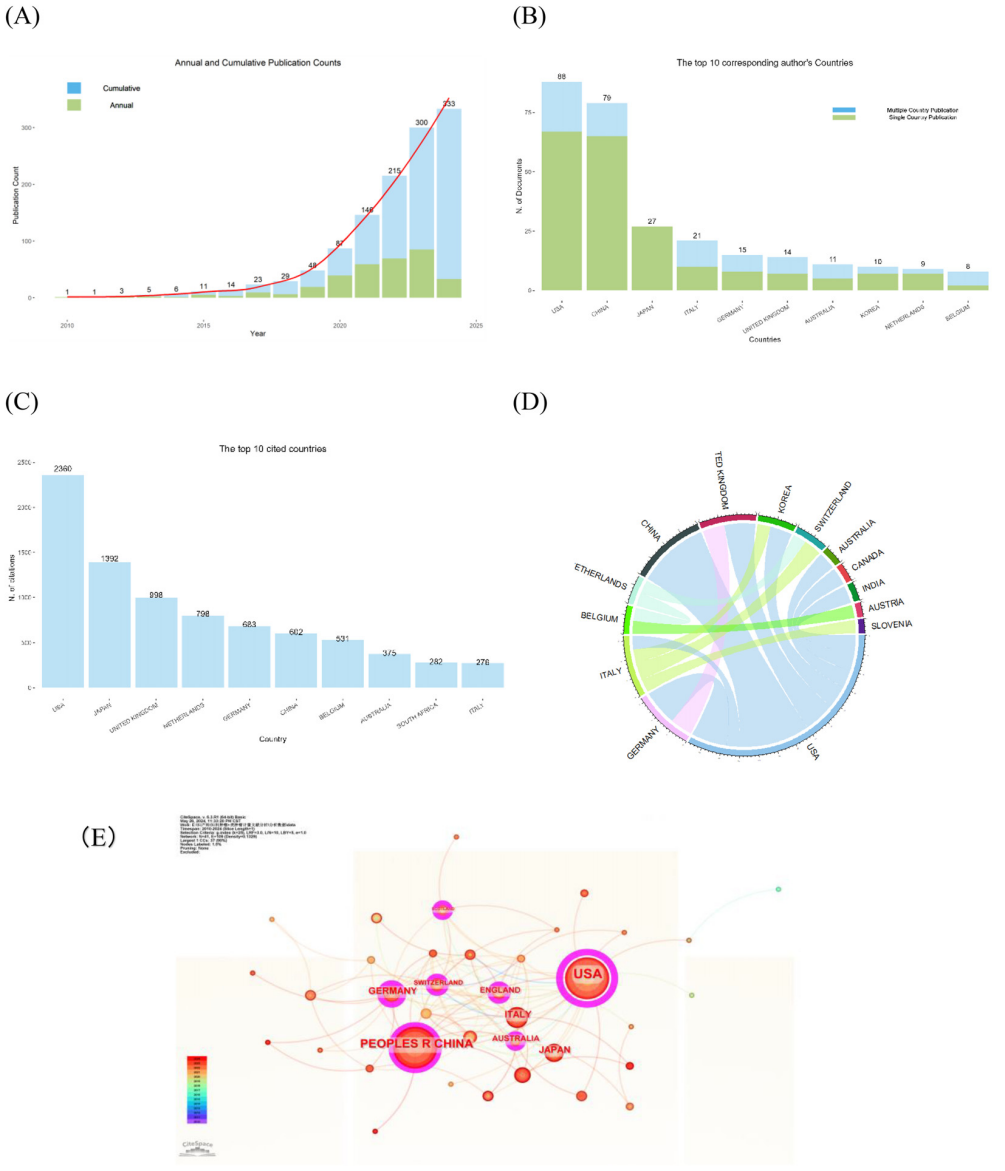
Global trends and country/region research contributions to organoids in GC. **(A)** Annual and Cumulative number of publications related to organoids in GC. **(B)** The top 10 Corresponding Author’s Countries **(C)** The top 10 Cited Countries/Region. **(D)** The international cooperation analysis (cooperation frequency > 2). The line between two countries/regions indicates cooperative relationship. **(E)** The network of countries/regions from CiteSpace. The size of the nodes represents the total number of publications for a country/region, with larger nodes indicating a greater number of articles, and the nodes have purple outer rings in the network representing high betweenness centrality which is the importance of location in a network of nodes.

According to the literature screening process (**Figure 1**), a total of 333 literatures were deemed suitable and included in the final analysis. From 2010 to 2024, the number of annual and cumulative publications was shown in **Figure 2A**. There was a significant increase in the number of publications from 2019 onwards. In 2018, there were less than 10 articles published per year, but this number rose to 85 articles in 2023. Furthermore, as of April 2024, 33 papers were published.

Based on the analysis of corresponding authors’ countries by Bibliometrix R Package, we found that the United States of America (USA) had the largest number of articles (n=88, 26.4%), followed by China (n=79, 23.7%), Japan (n=27, 8.1%), and Italy (n=21, 6.3%) (**Figure 2B**). In addition, USA also collaborated with other countries or regions frequently with 21 papers (23.9%). In the citation statistics, the USA had the highest number of citations (n=2360), followed by Japan (n=1392) and the United Kingdom (n=998) (**Figure 2C**). **Figure 2D** illustrates the collaboration status in organoid research for GC using a chord diagram. In **Figure 2E**, CiteSpace was utilized to analyze the national network map. There were seven countries or regions such as USA, China, Germany, Switzerland, England, Australia, and Scotland, which play the importance of roles in the research field.

### 3.2 Analysis of authors and institutions

**Figure 3:**
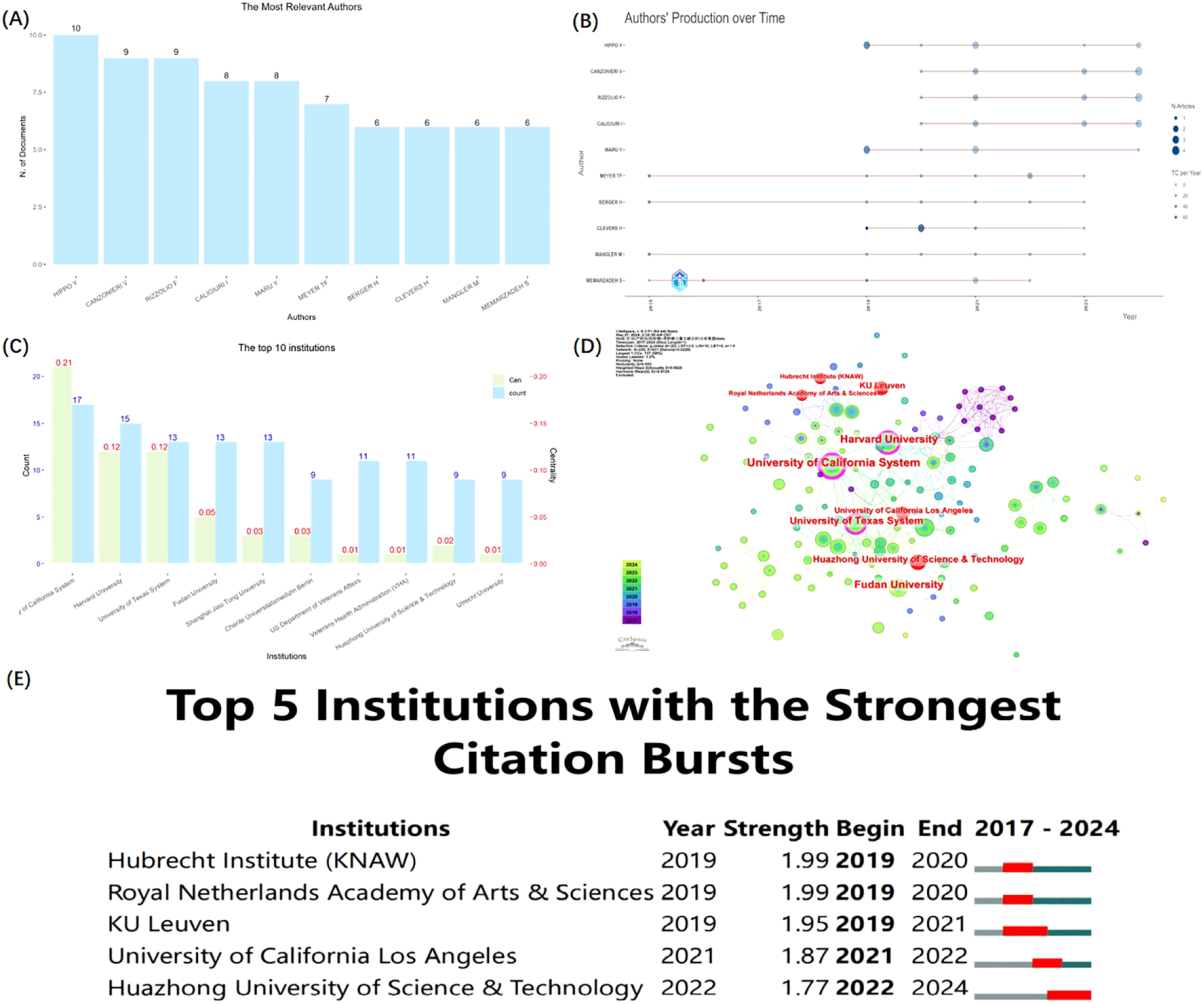
The distribution of authors and institutions in GC and organoids research. **(A, B)** The top 10 most relevant authors and authors’ production over time. **(C)** The top 10 institutions with counts and centrality. **(D)** The network of institutions from CiteSpace. **(E)** The Top 5 institutions with the strongest citation Bursts from CiteSpace.

There was a total of 2,621 authors in the field of GC organoids, of which the average number of co-authors per Document was 9.81. We found that the top 10 authors in this field have published 75 papers, which account for 22.5% of all publications (**Figure 3A**). Additionally, **Figure 3B** shows the production of the top 10 relevant authors over time. A total of 10 papers published in this field by Hippo Y, spanning from 2019 to 2024, and Canzonieri V and Rizzolio F are co-authors of 9 articles, spanning 2020-2024. As of April 2024, the top five authors collectively published 10 papers in 2024, which accounts for 30.3% of the total publications (n=33). (shown in **Table 1**).

**Table 1:** The articles by the top 5 relevant authors in 2024.

Author	year	TI	SO	DOI
HIPPO Y	2024	IDENTIFICATION OF TARGET CELLS OF HUMAN PAPILLOMAVIRUS 18 USING SQUAMOCOLUMNAR JUNCTION ORGANOIDS	CANCER SCIENCE	10.1111/cas.15988
	2024	ESTABLISHMENT AND CHARACTERIZATION OF MULTIPLE PATIENT-DERIVED ORGANOIDS FROM A CASE OF ADVANCED ENDOMETRIAL CANCER	HUMAN CELL	10.1007/s13577-024-01048-z
CANZONIERI V & RIZZOLIO F	2024	HUMAN OMENTAL MATURE ADIPOCYTES USED AS PACLITAXEL RESERVOIR FOR CELL-BASED THERAPY IN OVARIAN CANCER	ADVANCED HEALTHCARE MATERIALS	10.1002/adhm.202304206
	2024	COPPER NITROPRUSSIDE: AN INNOVATIVE APPROACH FOR TARGETED CANCER THERAPY VIA ROS MODULATION	BIOMEDICINE & PHARMACOTHERAPY	10.1016/j.biopha.2023.116017
	2024	SILVER NITROPRUSSIDE AS AN EFFICIENT CHEMODYNAMIC THERAPEUTIC AGENT AND A PEROXYNITRITE NANOGENERATOR FOR TARGETED CANCER THERAPIES	JOURNAL OF ADVANCED RESEARCH	10.1016/j.jare.2023.03.005
	2024	UNVEILING THE PROMISING ANTICANCER ACTIVITY OF PALLADIUM II-ARYL COMPLEXES BEARING DIPHOSPHINE LIGANDS: A STRUCTURE-ACTIVITY RELATIONSHIP ANALYSIS	DALTON TRANSACTIONS	10.1039/d4dt00919c
MARU Y	2024	ESTABLISHMENT AND CHARACTERIZATION OF MULTIPLE PATIENT-DERIVED ORGANOIDS FROM A CASE OF ADVANCED ENDOMETRIAL CANCER	HUMAN CELL	10.1007/s13577-024-01048-z
CALIGIURI I	2024	HUMAN OMENTAL MATURE ADIPOCYTES USED AS PACLITAXEL RESERVOIR FOR CELL-BASED THERAPY IN OVARIAN CANCER	ADVANCED HEALTHCARE MATERIALS	10.1002/adhm.202304206
	2024	COPPER NITROPRUSSIDE: AN INNOVATIVE APPROACH FOR TARGETED CANCER THERAPY VIA ROS MODULATION	BIOMEDICINE & PHARMACOTHERAPY	10.1016/j.biopha.2023.116017
	2024	SILVER NITROPRUSSIDE AS AN EFFICIENT CHEMODYNAMIC THERAPEUTIC AGENT AND A PEROXYNITRITE NANOGENERATOR FOR TARGETED CANCER THERAPIES	JOURNAL OF ADVANCED RESEARCH	10.1016/j.jare.2023.03.005

The top 10 related institutions with the largest number of publications extracted by CiteSpace (**Figure 3C**). The University of California system had the highest number of publications (n=17) and the highest centrality value (0.21). Harvard University ranked second in the number of published documents (n=15), and both Harvard University and the University of Texas system ranked second in centrality value (0.12). In the network of relevant institutions, as shown in **Figure 3D**, a high centrality value indicates high betweenness centrality with purple outer rings. Additionally, based on the strongest citation bursts analysis (γ=0.8), the 5 nodes are shown in red in the network, which are Hubrecht Institute (KNAW), Royal Netherlands Academy of Arts & Sciences, KU Leuven, University of California Los Angeles, and Huazhong University of Science and Technology (HUST) (**Figure 3E**). What’s more, the citation bursts of HUST occurred in 2022-2024, indicating outstanding research in the field recently.

### 3.3 Analysis of active journals

**Figure 4:**
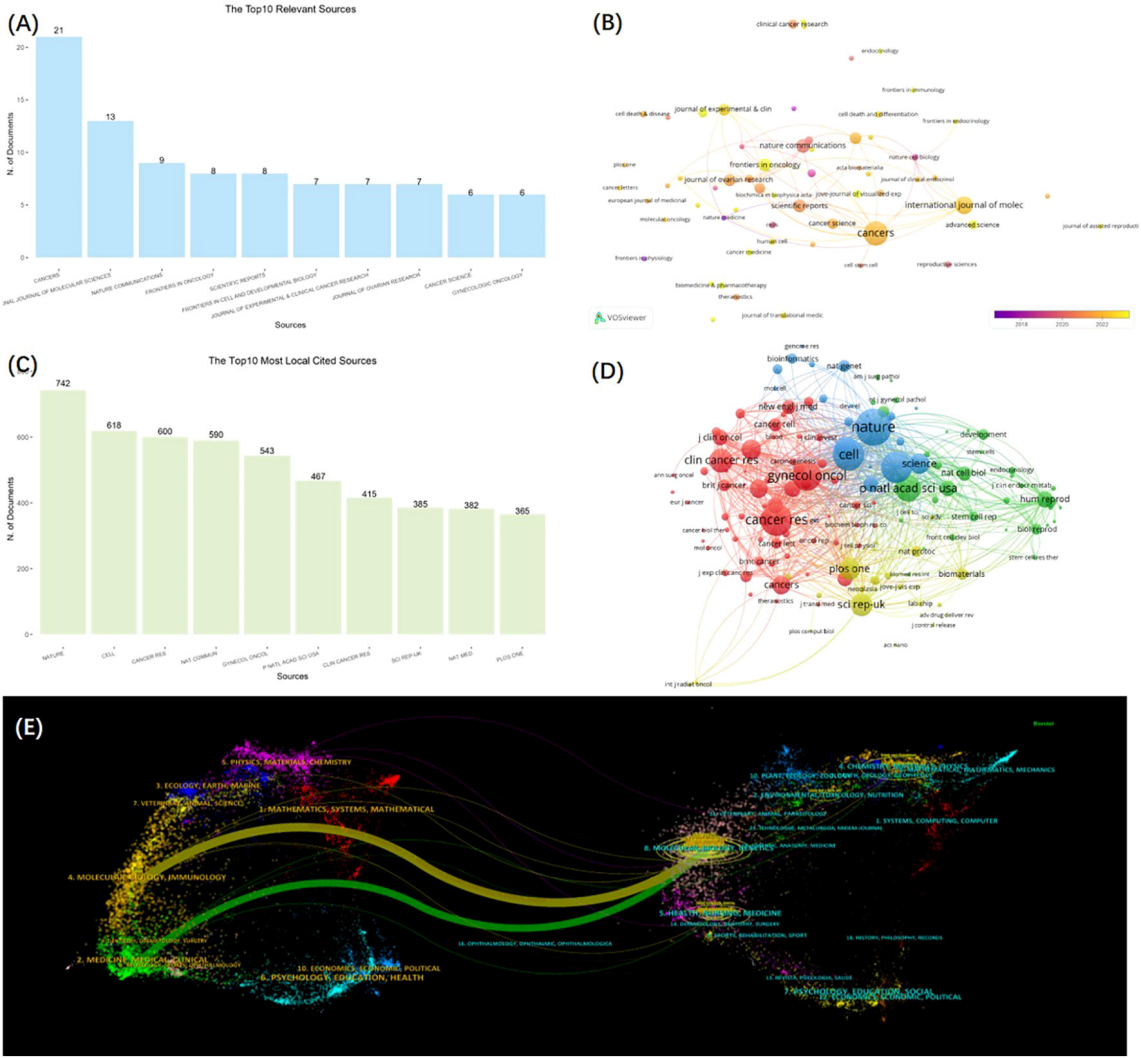
The distribution of authors and institutions in GC and organoids research. **(A)** The Top 10 most relevant sources. (B) Network visualization of sources analysis from VOSviewer. **(C)** The top 10 most local cited sources. **(D)** Network visualization of cited sources analysis from VOSviewer. **(E)** Dual-map overlay of journals from CiteSpace.

The outcome in the research field of ​GC organoids were published in 169 journals. *CANCERS* ranked first (n=21), followed by *INTERNATIONAL JOURNAL OF MOLECULAR SCIENCES* (n=13), *NATURE COMMUNICATIONS* (n=9), *FRONTIERS IN ONCOLOGY* (n=8), and *SCIENTIFIC REPORTS* (n=8), as shown in **Figure 4A**. Meanwhile, **Figure 4B** is a network visualization of source analysis by VOSviewer. The relevant information of the top 10 key journals in this field is presented in **Table 2**.

**Table 2:** The Top 10 most relevant journals.

Element	h_index	g_index	IF(2023)	TC	NP	PY_start
CANCERS	8	15	4.50	227	21	2018
INTERNATIONAL JOURNAL OF MOLECULAR SCIENCES	4	13	4.90	363	13	2017
NATURE COMMUNICATIONS	7	9	14.70	722	9	2015
SCIENTIFIC REPORTS	6	8	3.80	166	8	2017
FRONTIERS IN ONCOLOGY	3	4	3.50	23	8	2022
FRONTIERS IN CELL AND DEVELOPMENTAL BIOLOGY	5	7	4.60	77	7	2020
JOURNAL OF EXPERIMENTAL & CLINICAL CANCER RESEARCH	4	6	11.40	45	7	2021
JOURNAL OF OVARIAN RESEARCH	3	7	3.80	52	7	2013
GYNECOLOGIC ONCOLOGY	4	6	4.50	192	6	2019
CANCER SCIENCE	3	6	5.70	375	6	2017

Then, we further analyzed the local cited source in the field and presented the top 10 major journals using a bar chart (**Figure 4C**) and the network of cited journals through VOSviewer analysis (**Figure 4D**). Rounding out the top 5 journals were *NATURE* (n = 742), *CELL* (n = 618), *CANCER RESEARCH* (n = 600), *NATURE COMMUNICATIONS* (n = 590), and *GYNECOLOGIC ONCOLOGY* (n = 543).

**Figure 4E** depicts the Dual-map overlay of journals by CiteSpace. The citing journals are on the left, the cited journals on the right, and the curve lines represent the citation path association of journals. Tow citation paths​ (orange and green) were recognized, indicating that the studies published in Molecular/Biology/Immunology journals (orange path) and Medicine/Medical/Clinical journals (green path) were cited by the research published in Molecular/Biology/Genetics journals.

### 3.4 Analysis of keywords

**Figure 5:**
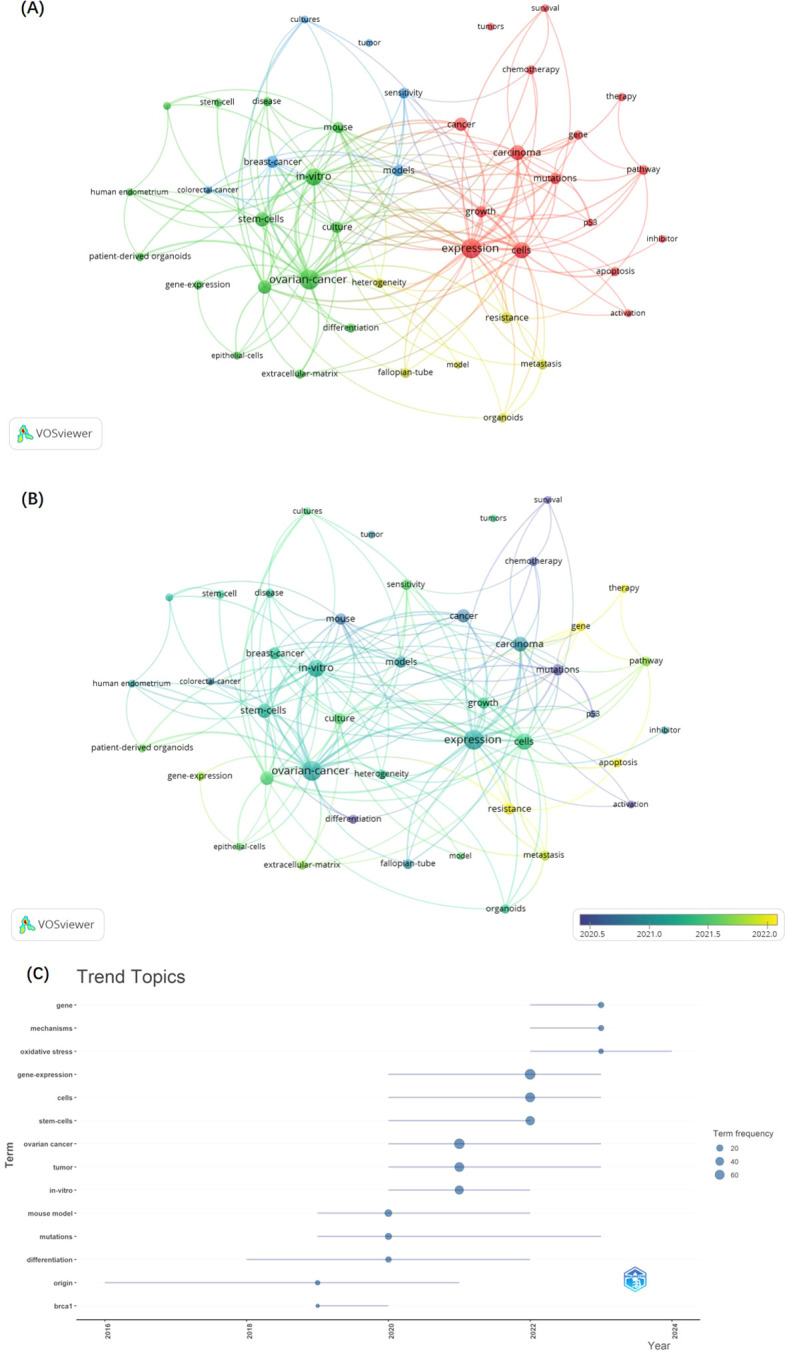
The network of keywords plus co-occurrence. **(A)** Overlay map of keyword according to clusters from VOSviewer. **(B)** The timestamp visualization of keywords from VOSviewer. **(C)** the trend topics of KeywordPlus from Bibliometrix R Package.

VOSviewer software was employed to construct a keyword co-occurrence network, facilitating the identification of emerging trends and hotspots within the research landscape of GC and organoids, as illustrated in **Figure 5**. This network analysis elucidated the interconnections and prominence of various research themes, offering a visual synopsis of the field’s intellectual structure and dynamics. We set the minimum number of keywords plus occurrences to 10, and 43 keywords plus meet the threshold. The nodes and font sizes in **Figure 5A** represent the frequency of the keywords and their importance in the field. The keywords were divided into 4 clusters.

Cluster 1 includes keywords plus such as activation, apoptosis, cancer, carcinoma, cells, chemotherapy, expression, gene, growth, inhibitor, mutations, p53, pathway, survival, therapy, and tumors, indicating that this cluster may be related to the molecular and cellular mechanisms of cancer development and treatment.

In cluster 2, keywords such as culture, differentiation, disease, epithelial-cells, extracellular-matrix, gene-expression, human endometrium, *in-vitro*, long-term, long-term expansion, mouse, ovarian-cancer, patient-derived organoids, and stem-cells indicate that the text in this section focuses on issues related to *in vitro* cell biology and the study of organoids for modeling diseases and their long-term behavior.

Cluster 3 keywords included breast-cancer, colorectal-cancer, cultures, models, sensitivity, and tumor, suggesting that the texts represented by this cluster may be more focused on the development and application of cancer models for assessing drug sensitivity and therapeutic strategies.

Cluster 4 contains many cancer progression and heterogeneity keywords, such as fallopian-tube, heterogeneity, metastasis, model, organoids, and resistance, indicating that the text represented by this cluster is focused on the study of cancer metastasis, the use of organoids to model complex tumor characteristics, and the development of drug resistance.

As shown in **Figure 5B**, the keywords appearing in recent years, such as gene, therapy, apoptosis, resistance, and metastasis, were all related to advances in cancer development mechanistic and the challenges of drug resistance. The keywords analysis conducted using the Bibliometrix R Package, specifically the trend topics of keywords Plus (**Figure 5C**), corroborates the same conclusion. The focus within the trend topics has been on the most recent three keywords: gene, mechanisms, and oxidative stress, which are linked to the mechanistic research. Besides, the field of oncology research has been the focus of scholars and organoids as the pre-clinical models is associated with good research prospects.

### 3.5 Analysis of references and documents

**Figure 6:**
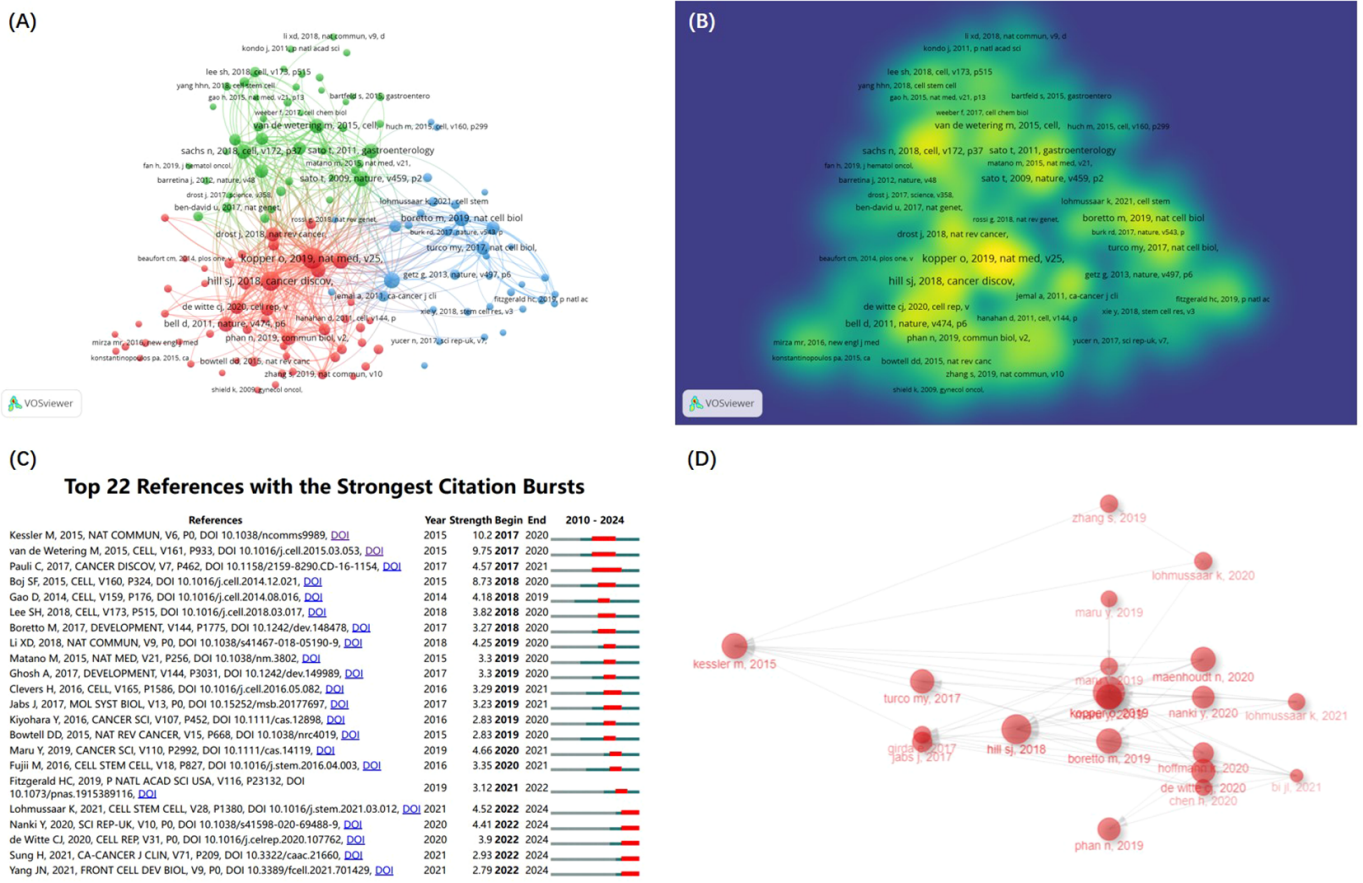
The references and documents analysis. **(A)** The co-citation network of references according to clusters from VOSviewer. **(B)** Density map of cocited analysis of references from VOSviewer. **(C)** Visualization map of the top 22 references with the strongest citation bursts from CiteSpace. **(D)** the Historiograph of the GC and organoids research from Bibliometrix R Package.

A total of 18,205 references in the research field were cited. The co-citation network of 134 references cited over 10 times (**Figure 6A**) is constructed by the VOSviewer. The intensity reference citations are shown in **Figure 6B**, with brighter colours indicating more frequent citations.

The burst detection algorithm invented by Kleinberg (16) of CiteSpace visualize the top 22 references with the strongest citation bursts (**Figure 6C**). There are 5 references with the citation bursts end in 2024 that reflect the most important reference in the field and identify emerging topics and research frontiers (9). Among the 5 references, 3 were associated with OC organoids research, and 1 CC.

**Table 3** lists the top 10 cited references that form the foundation of GC organoids research. Among these 10 articles, 9 were associated with organoids research, and 4 were from the OC research.

**Table 3:** The top 10 most cited references.

cited reference	citations	total link strength
kopper o, 2019, nat med, v25, p838, doi 10.1038/s41591-019-0422-6	106	325
hill sj, 2018, cancer discov, v8, p1404, doi 10.1158/2159-8290.cd-18-0474	85	267
kessler m, 2015, nat commun, v6, doi 10.1038/ncomms9989	56	182
maru y, 2019, gynecol oncol, v154, p189, doi 10.1016/j.ygyno.2019.05.005	56	216
boretto m, 2019, nat cell biol, v21, p1041, doi 10.1038/s41556-019-0360-z	54	148
bell d, 2011, nature, v474, p609, doi 10.1038/nature10166	53	142
sato t, 2009, nature, v459, p262, doi 10.1038/nature07935	52	166
maenhoudt n, 2020, stem cell rep, v14, p717, doi 10.1016/j.stemcr.2020.03.004	51	202
sachs n, 2018, cell, v172, p373, doi 10.1016/j.cell.2017.11.010	51	180
van de wetering m, 2015, cell, v161, p933, doi 10.1016/j.cell.2015.03.053	49	160

In addition, the top 10 local cited documents is listed in **Table 4** that also represent the most important research literature in the field. what’s more, based on the Bibliometrix R Package, we obtained the Historiograph map, featuring 20 pivotal, highly-cited documents in the GC and organoids (**Figure 6D**), which afford a general overview of the progress and knowledge base. This field mainly studies the establishment of organoids, their advantages as preclinical models and drug screening, and is more interested in OC.

**Table 4:** The top 10 most global cited documents.

Document	DOI	Year	Local Citations	Global Citations
KOPPER O, 2019, NAT MED	10.1038/s41591-019-0422-6	2019	106	428
HILL SJ, 2018, CANCER DISCOV	10.1158/2159-8290.CD-18-0474	2018	85	277
KESSLER M, 2015, NAT COMMUN	10.1038/ncomms9989	2015	56	297
MARU Y, 2019, GYNECOL ONCOL	10.1016/j.ygyno.2019.05.005	2019	56	98
BORETTO M, 2019, NAT CELL BIOL	10.1038/s41556-019-0360-z	2019	54	253
MAENHOUDT N, 2020, STEM CELL REP	10.1016/j.stemcr.2020.03.004	2020	51	89
TURCO MY, 2017, NAT CELL BIOL	10.1038/ncb3516	2017	47	379
DE WITTE CJ, 2020, CELL REP	10.1016/j.celrep.2020.107762	2020	46	138
PHAN N, 2019, COMMUN BIOL	10.1038/s42003-019-0305-x	2019	41	156
NANKI Y, 2020, SCI REP-UK	10.1038/s41598-020-69488-9	2020	39	78

## 4 Discussion

### 4.1 General information

This study conducted a comprehensive bibliometric analysis of the literature in the research of GC and organoids, utilizing advanced analytical and visualization tools such as CiteSpace, VOSviewer, and the Bibliometrix R Package. The analysis encompassed global research trends, including the number of publications, contributing countries or regions, institutions, authors, journals, keywords, and references. A total of 333 documents were retrieved from the WoSCC, spanning the period from 2010 to 2024, and these literatures were disseminated across 169 journals by 2,621 authors affiliated with 681 institutions in 41 countries or regions.

The bibliometric analysis revealed a marked increase in the number of publications on GC and organoids since 2019. A statistical examination of publication outputs by various countries and institutions has identified leading nations and research entities with significant contributions to the field. The US, China, and Japan have emerged as major contributors to organoids research in GC, and US, Japan, and the United Kingdom demonstrate advanced maturity in this domain. CiteSpace analysis identified the top 10 institutions with five from the United States, three from China, one from the Netherlands, and one from Germany. Notably, the University of California exhibited the highest count and betweenness centrality values, indicating its pivotal role. Enhanced collaboration and communication among these entities are essential for surmounting academic silos and propelling forward the field of GC and organoids research.

In the hierarchy of the most relevant authors in the field of GC organoids, Dr. Hippo Y leads with the highest publication count (10, 3%), followed by Drs. Canzonieri V (9, 2.7%) and Rizzolio F (9, 2.7%). In recent years, they continued to research in the field, and 6 articles have been published by them in 2024(n=33). Despite this, the author analysis by CiteSpace did not identify any author with a centrality score above the threshold of 0.10, denoting no authors with significant influence in the field.

Through an analysis of the publication outlets, it has been determined that the journal with the highest number of publications in the field of GC and organoids is *CANCERS*, accounting for 21 articles (6.3%). Among the TOP10 Most Relevant Sources, two journals have an impact factor exceeding 10 for the year 2023: *NATURE COMMUNICATIONS* with an impact factor of 14.7 and *JOURNAL OF EXPERIMENTAL & CLINICAL CANCER RESEARCH* 11.4. In the citation analysis, the most frequently cited sources are *NATURE* with 742 citations, *CELL* with 618, and *CANCER RESEARCH* with 600. The distribution of sources and citations is instrumental in identifying the core journals for publishing research related to GC organoids, thereby aiding scholars in establishing scientific achievements. These data will be valuable for future scholars when selecting appropriate journals for the submission of related manuscripts.

### 4.2 Analysis of key documents

To rapidly grasp the key documents in GC Organoids field, we can utilize the Bibliometrix-R-package for Document analysis to construct a Historiograph (**Figure 6D**) that includes the top 20 documents with the most local citations in this field. Analysis of the key documents reveals that the primary focus has been on three aspects: the construction of organoids, their characteristics, and their application as clinical models.

#### 4.2.1 Construction of gynecological cancer organoids

GC organoids possess advantages such as high success rates, low costs, and the preservation of tumor heterogeneity (17). They can also maintain long-term propagation (18). Most importantly, organoids can be successfully established in short periods (19–23), and the tissues required for their creation are easily accessible. In addition to the primary tumor, they can also be derived from malignant effusions (21) and cervical brushings (24). In the research on the construction process of organoids, signals that affect the continuous expansion of organoids include Wnt (25, 26) and Notch (26), and NRG1 is identified as a key factor for the growth of OC organoids (27).

Researchers have improved organoid generation and assessment techniques in recent year. Matrigel bilayer organoid culture (MBOC) protocol can enhance the efficiency of organoid culture (28). Organoids via a 3D ring-shaped model enable rapid high-throughput screenings and preserving original histological features, which are suitable for clinical decision-making (20). The DeathPro, an automated microscopy-based assay, can identify cell death induced by drugs and inhibition of proliferation, elucidating genotype-drug sensitivity correlations (29).

#### 4.2.2 Characteristics of GC organoids

It has been found that organoids replicate the histological, genomic, and mutational characteristics of the original tumor or tissue (18, 19, 25, 27, 28, 30–34), while also exhibiting genetic diversity and heterogeneity both intra- and inter-patient (33). The heterogeneity between patients (32) aids in using organoids as ideal models for personalized therapy. Diverse organoids clone derived from distinct tumor regions highlight inter-clonal heterogeneity within patients (28), which contributes to the study of tumor complexity. Moreover, studies have shown that Organoids display inter- and intrapatient drug response heterogeneity, partly explained by genetic aberrations (22).

#### 4.2.3 Application of GC organoids

Organoids serve as valuable clinical models in drug screening, effectively emulating the reactions of original tumor (19–22, 27, 28, 30, 32–34). They also function as clinical models to research tumor origin (35, 36). Furthermore, organoids are utilized as clinical models for genetic manipulation and other related research. Hill, SJ, et al. employed organoids derived from OC to assess DNA repair and evaluate treatment sensitivity (31). Lohmussaar et al. suggested that healthy ecto- and endocervical 3D organoid cultures could open exciting new avenues for studying CC and infections (24).

The key documents analysis helps researchers quickly understand the key points in the research field of GC organoids.

### 4.3 Hotspots and frontiers

In bibliometric analysis, through keyword co-occurrence and clustering analysis, trends in research themes and current hotspots are identified (37, 38). In the GC organoids field, the current hotspots are the use of organoids as clinical models to investigate tumorigenesis and drug resistance mechanisms. As a model for GC research, organoids are extensively utilized in OC, CC, and EC. Notably, organoids of OC have been particularly well-studied, with prominent research hotspots. OC emerges as one of the important keywords in this analysis. Thus, the following discussion will center on OC organoids to explore the current research frontiers and hotspots in this field.

Organoids, as tumor research models, can more accurately simulate *in-vivo* tumor growth (39). They not only faithfully represent the pathological and genetic characteristics of the original tumor (40), but also, through the diversity of their genetic features and the application of genetic manipulation techniques, provide a valuable platform for research into tumor mechanisms and drug development. Next, we will discuss the current state of research in two aspects: the diversity of genetic characteristics in organoids and the alteration of gene expression.

#### 4.3.1 Diversity of genetic characteristics in organoids

By comparing the diversity of Organoids and integrating this with clinical information and drug response features of patients, it becomes easier to explore the complex mechanisms of action and resistance analysis of tumors. Al-Alem et al. constructed patient-derived organoids (PDOs) from four different patients as experimental models and found that different organoids exhibited dose-dependent responses to anti-Sialyl Tn antibody-drug conjugate (anti-STn-ADC), explained by the different level of Sialyl Tn(STn) (41). Miao et al. grouped PDOs based on different Ferroptosis suppressor protein 1 (FSP1) expression levels and discovered that FSP1 is a prognostic indicator for OC patients, and that olaparib and iFSP1 (a FSP1 inhibitor) inhibit the proliferation of OC-PDOs, but the effect is not due to ferroptosis (42). Gao et al. validated the drug effect by grouping PDOs based on the glutamine metabolism prognostic index (GMPI) and found that GMPI can serve as a reliable prognostic indicator for patients and help optimize treatment strategies (43). Cai et al. found that organoids derived from different patients exhibited different drug responses (resistant or sensitive to cisplatin), and further analysis of the different drug response groups revealed differences in senescence-associated genes (SGK1, VEGFA) (44). Trillsch et al. suggested that organoids constructed from different disease stages (untreated primary tumors, advanced tumors, recurrent tumors) provide the possibility for direct comparative analysis (45). Organoids can be generated from cells that are poorly represented in the biopsy, thereby enabling the selection of particular tumor molecular traits (40), which further enriches the genetic phenotype diversity of organoids and offers more possibilities for the study of related mechanisms.

#### 4.3.2 Modulating gene expression in organoids

In the investigation of their mechanisms, it is possible not only to study via the diversity of genes expression within Organoids but also to manipulate the expression of specific genes through genetic engineering techniques to observe their effects on pathogenesis and drug sensitivity. Zhang et al. constructed organoids resistant to platinum-based drugs from OC tumor cells derived from ascites, and by combining use of si-ZDHHC12 and cisplatin, they observed changes in the drug resistance of organoids while affecting the expression level of ZDHHC12, finding that the inhibition of ZDHHC12 can enhance the antitumor activity of cisplatin in OC (46). Wang et al., to explore the mechanism of circ-RAD23B in organoids, controlled the expression level of circ-RAD23B by transfecting pLV-circRAD23B and sh-circRAD23B into PDOs, and observed the proliferative capacity, apoptosis levels, and carboplatin resistance of PDOs, showing that the level of circRAD23B expression can modulate the sensitivity or resistance of PDOs to carboplatin (39). Dai et al. created organoids from primary human normal fallopian tube epithelial (FTE) and introduced TP53 and RAD51D knockdown organoids to explore their impact on mutations arising from FTE damage, emphasizing that the use of FTE organoids with RAD51D mutations can serve as a valuable *in-vitro* platform for early detection of carcinogenic effects, mechanism exploration, and drug screening (47). Gong et al. discovered that different sources of PDOs showed varying chemosensitivity to epithelial ovarian cancer (EOC), and that Zinc finger SWIM-type containing 4 (ZSWIM4) inhibition can enhance the chemosensitivity of EOC cells by improving intracellular glycine metabolism reprogramming (48).

OC, particularly high-grade serous carcinoma (HGSOC), continues to pose a significant threat to public health, with BRCA mutations and Homologous Recombination (HRD) status being pivotal in the diagnostic and therapeutic for OC (49). Organoids, when employed as clinical models for HRD assessment, contribute significantly to the prediction of patient response to therapy (49, 50). PDOs offer a treasure trove of opportunities for exploring tumor mechanisms and drug research. Despite the current limitations associated with the lack of a tumor microenvironment in organoids (8), there is considerable anticipation for the potential of organoids in cancer research. Organoids are regarded as promising tools for precision oncology and are seen as invaluable platforms for biological and pharmacological research (40, 51).

### 4.4 Limitations

​Firstly, this study retrieved documents from the WoSCC database with restrictions on document types and languages, which may have resulted in the exclusion of some relevant literature. Additionally, while some documents were included based on keyword searches that met the retrieval criteria, there is a possibility that documents whose content does not align with the research topic were still included. Therefore, it is essential to establish a rigorous search strategy to ensure the comprehensiveness and reliability of the input data. Secondly, although bibliometric software such as CiteSpace and VOSViewer can quickly analyze relevant literature, they provide only metadata rather than the full text, which may lead to biases or omissions in the analysis results. Thirdly, given the continuous updating of databases, both the number of retrieved documents and the citation counts of obtained documents are in a state of constant flux, which may result in discrepancies between the results and the most recent research. These limitations may slightly affect the overall results; however, the primary objective of bibliometric analysis is to focus on the main trends and hotspots in a field through the analysis of a large volume of literature data. Overall, our study provides a foundation for understanding the research themes, hotspots, and development trends of organoids and ​GC.

## 5 Conclusion

​Through a detailed bibliometric analysis of Organoids and ​GC, this study assessed the literature information across various years, countries, institutions, authors, disciplines, and journals, and analyzed the development of topics and potential future research hotspots. Our research observed that the field began to receive focused attention in 2019, with a rapid increase in research outcomes. Our study provides fundamental information on research in this field and identifies potential collaborators for interested researchers. In this domain, Organoids, as *in vitro* models for ​GC research, not only allow for the establishment of models with a small amount of tissue in a short time but also stably replicate the histological and genetic characteristics of the original tumor. They are manipulable for exploring the mechanisms of disease occurrence and progression. Additionally, as preclinical models, they provide compelling evidence for personalized therapy and prediction of patient drug responses.

## Data Availability

The original contributions presented in the study are included in the article/supplementary material. Further inquiries can be directed to the corresponding author.
